# Inosine reverses multidrug resistance in Gram-negative bacteria carrying mobilized RND-type efflux pump gene cluster *tmexCD-toprJ*

**DOI:** 10.1128/msystems.00797-24

**Published:** 2024-09-10

**Authors:** Fulei Li, Tianqi Xu, Dan Fang, Zhiqiang Wang, Yuan Liu

**Affiliations:** 1 Jiangsu Co-innovation Center for Prevention and Control of Important Animal Infectious Diseases and Zoonoses, College of Veterinary Medicine, Yangzhou University, Yangzhou, China; 2 Joint International Research Laboratory of Agriculture and Agri-Product Safety, the Ministry of Education of China, Yangzhou University, Yangzhou, China; 3 Institute of Comparative Medicine, Yangzhou University, Yangzhou, China; Institut de Recherche pour le Developement Delegation Regionale Occitanie Centre de Documentation, Montpellier, France

**Keywords:** antibiotic, antimicrobial resistance, inosine, metabolism, bacteria

## Abstract

Antimicrobial resistance is rapidly increasing worldwide, highlighting the urgent need for pharmaceutical and nonpharmaceutical interventions to tackle different-to-treat bacterial infections. Tigecycline, a semi-synthesis glycylcycline for parenteral administration, is widely recognized as one of the few effective therapies available against pan-drug resistant Gram-negative pathogens. Regrettably, the efficacy of multiple drugs, including tigecycline, is currently being undermined due to the emergence of a recently discovered mobilized resistance-nodulation-division-type efflux pump gene cluster *tmexCD1-toprJ1*. Herein, by employing untargeted metabolomic approaches, we reveal that the expression of *tmexCD1-toprJ1* disrupts bacterial purine metabolism, with inosine being identified as a crucial biomarker. Notably, the supplementation of inosine effectively reverses tigecycline resistance in *tmexCD1-toprJ1*-positive bacteria. Mechanistically, exogenous inosine enhanced bacterial proton motive force, which promotes the uptake of tigecycline. Furthermore, inosine enhances succinate biosynthesis by stimulating the tricarboxylic acid cycle. Succinate interacts with the two-component system EnvZ/OmpR and upregulates OmpK 36, thereby promoting the influx of tigecycline. These actions collectively lead to the increased intracellular accumulation of tigecycline. Overall, our study offers a distinct combinational strategy to manage infections caused by *tmexCD-toprJ*-positive bacteria.

**IMPORTANCE:**

TMexCD1-TOprJ1, a mobilized resistance-nodulation-division-type efflux pump, confers phenotypic resistance to multiple classes of antibiotics. Nowadays, *tmexCD-toprJ* has disseminated among diverse species of clinical pathogens, exacerbating the need for novel anti-infective strategies. In this study, we report that *tmexCD1-toprJ1*-negative and -positive bacteria exhibit significantly different metabolic flux and characteristics, especially in purine metabolism. Intriguingly, the addition of inosine, a purine metabolite, effectively restores the antibacterial activity of tigecycline by promoting antibiotic uptake. Our findings highlight the correlation between bacterial mechanism and antibiotic resistance, and offer a distinct approach to overcome *tmexCD-toprJ*-mediated multidrug resistance.

## INTRODUCTION

Since the discovery of penicillin at the beginning of the 20th century, antibiotics have revolutionized contemporary healthcare by saving numerous individuals from dangerous situations (1). However, the emergence and prevalence of antimicrobial resistance in pathogenic bacteria, especially in Gram-negative bacteria, result in increased mortality and higher medical costs, posing a global burden to public health (2). *Klebsiella pneumoniae*, a bacterium belonging to the Enterobacteriaceae family, is implicated in various infectious diseases such as urinary tract infections, bacteremia, pneumonia, and liver abscesses (3, 4).

Tigecycline, a glycylcycline antibiotic, hinders bacterial protein synthesis by binding to the 30S ribosomal subunit and blocking tRNA entry (5). Nowadays, tigecycline is recognized as one of the last-line antibiotics against different-to-treat Gram-negative bacterial infections, whereas the acquisition of drug resistance severely compromises its clinical effectiveness (6). It has been reported that the mechanism of tigecycline resistance is mainly mediated by efflux pumps and regulatory factors. The resistance-nodulation-cell division (RND)-type transporters, particularly the AdeABC, AdeFGH, and AdeIJK efflux pumps, are associated with tigecycline resistance (7). These pumps were commonly encoded on the chromosome, although a limited number of studies have reported on plasmid-borne elements (8). In 2020, a new plasmid-borne gene cluster called *tmexCD1-toprJ1* was discovered, which confers bacterial resistance to various types of antibiotics, including cephalosporins, phenicols, quinolones, and tetracyclines (9, 10). Nowadays, *tmexCD-toprJ* gene and its variants have been reported worldwide (11, 12). Therefore, there is an urgent need to explore effective strategies for combating *tmexCD-toprJ*-positive bacterial infections.

While the targets and mechanisms of action for conventional antibiotics have been extensively studied, there is an increasing recognition that bacterial metabolism is also closely associated with antibiotic efficacy (13). Specifically, antibiotics alter bacterial metabolic state, which also, in turn, affects bacterial susceptibility to antibiotics (14–16). Consistent with these opinions, in the context of several experimental scenarios, a series of evidence indicates that metabolic reprogramming by the supplementation of exogenous metabolites provides a distinct approach to reverting antibiotic resistance and enhancing antibiotic activity (17–19). The rationale behind this strategy is that bacteria downshift certain metabolic pathways upon gaining antibiotic resistance, which can be rescued by the supplementation of exogenous metabolites (20, 21). One of the underlying mechanisms is dependent on the activation of the pyruvate cycle, which in turn increases the production of the reduced form of nicotinamide adenine dinucleotide (NADH) and proton motive force (PMF) and stimulates the uptake of antibiotic (21). Despite these intensive efforts, the impact of mobilized RND-type efflux pump gene cluster *tmexCD-toprJ* on the metabolic state of bacteria and its clinical implications were still not fully understood.

In this study, we compared the metabolomes of *tmexCD1-toprJ1*-negative and -positive bacteria to investigate the metabolic signatures of tigecycline-resistant bacteria. Notably, purine metabolism was greatly suppressed in *tmexCD1-toprJ1*-positive bacteria. Conversely, the addition of inosine, an intermediate compound in purine metabolism, remarkably restored the susceptibility of *tmexCD1-toprJ1*-positive strains to tigecycline. The underlying mechanism is that exogenous inosine promoted the tricarboxylic acid (TCA) cycle by substrate activation, which in turn increased the production of succinate. Meanwhile, succinate enhanced the expression of OmpK 36 and stimulated the uptake of tigecycline via EnvZ/OmpR. Our work underscores the value of metabolic reprogramming in overcoming antibiotic resistance.

## RESULTS

### The expression of *tmexCD1-toprJ1* results in bacterial metabolomic shift

**Fig 1:**
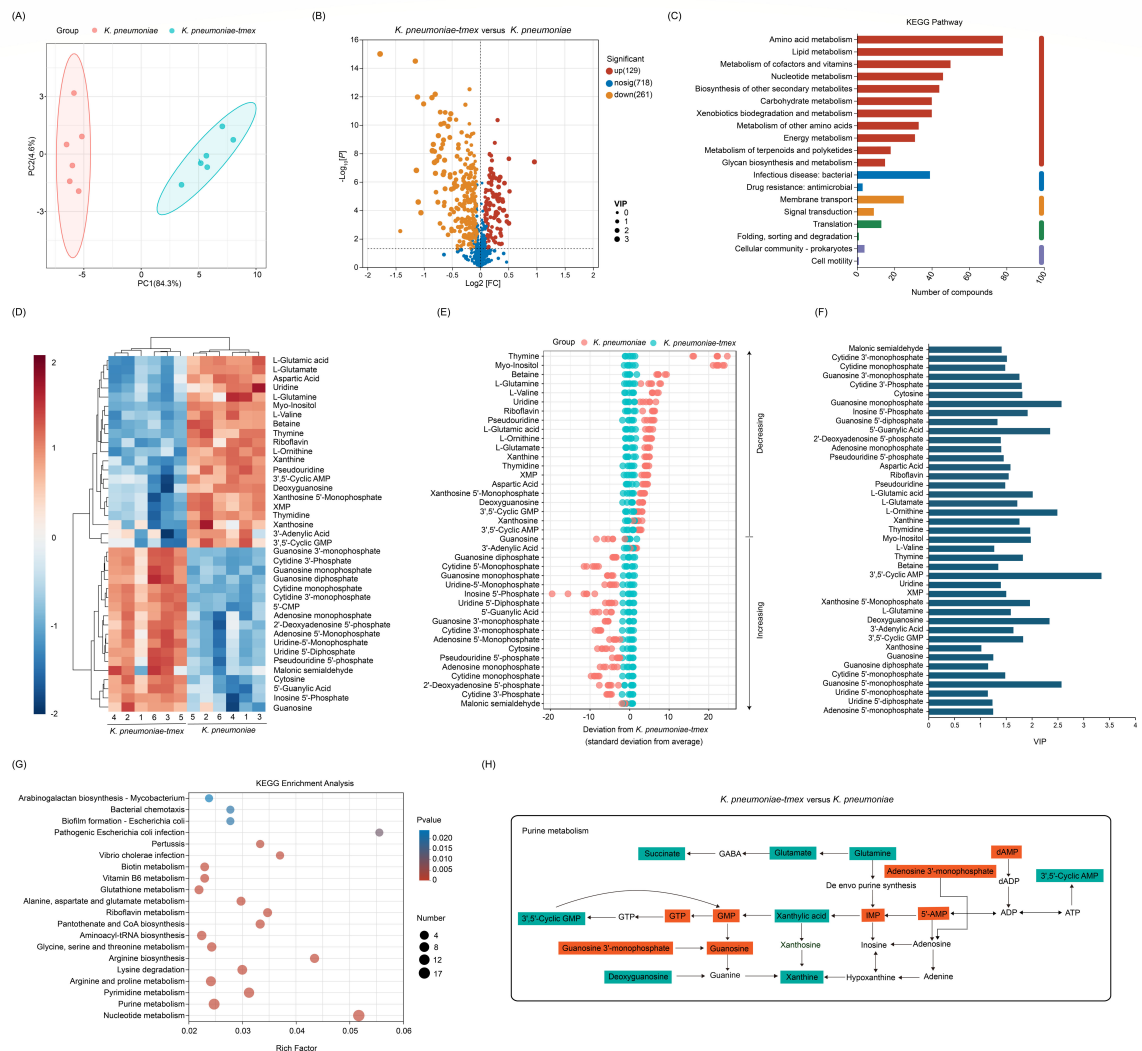
Metabolomic profiling of *tmexCD1-toprJ1*-negative and -positive *K. pneumoniae*. (**A**) PCA analysis of 390 metabolites identifies components 1 and 2 as determinants of variability in metabolite abundance between *tmexCD1-toprJ1*-negative and -positive *K. pneumoniae* K6. (**B**) Volcano plot of metabolome analysis of *tmexCD1-toprJ1*-negative and -positive *K. pneumoniae* K6. (**C**) Number of compounds enriched in KEGG pathways. (**D**) Heatmap showing relative abundance of differential metabolites in *K. pneumoniae* K6*/*pUC19*-tmexCD1-toprJ1* compared with *K. pneumoniae* K6. Blue to orange colors correspond to low to high abundance. XMP, xanthosine monophosphate; 5′-CMP, Cytidine 5′-Monophosphate. (**E**) *Z* scores (standard deviation from average) correspond to the data in (**D**). Each point represents one technical repeat in one metabolite. Green, *tmexCD1-toprJ1*-negative *K. pneumoniae*; Orange, *tmexCD1-toprJ1*-positive *K. pneumoniae*. (**F**) The metabolite variable importance in the projection value (VIP), which indicates the contribution of the metabolite to the difference between *tmexCD1-toprJ1*-negative and -positive *K. pneumoniae*. (**G**) Enriched pathways in *K. pneumoniae* K6*/*pUC19*-tmexCD1-toprJ1*. (**H**) Metabolic networks of purine biosynthesis.

To explore the impact of *tmexCD1-toprJ1* expression on bacterial metabolomic state, *tmexCD1-toprJ1* gene was cloned into a high-copy plasmid, pUC19, to generate pUC19-*tmexCD1-toprJ1*. An empty vector without *tmexCD1-toprJ1* (pUC19) was included as the control. Subsequently, pUC19-*tmexCD1-toprJ1* and pUC19 plasmids were transformed into two reference strains, *K. pneumoniae* K6 and *Escherichia coli* DH5α, respectively. Antimicrobial susceptibility test results indicated that the minimal inhibitory concentration (MIC) values of *K. pneumoniae* K6/pUC19-*tmexCD1-toprJ1* and *E. coli* DH5α/pUC19-*tmexCD1-toprJ1* to tigecycline were increased by 16-fold and 32-fold, respectively (Table S1), suggesting that *tmexCD1-toprJ1* was successfully expressed. Then, we performed metabolomic profiling of *tmexCD1-toprJ1*-negative and -positive bacteria using liquid chromatography-mass spectrometry (LC-MS). Unsupervised principal component analysis (PCA) revealed that *tmexCD1-toprJ1* systemically altered the metabolites environment (Fig. 1A; Fig. S1A). These differentially expressed metabolites (DEMs) were plotted in volcanoes (Fig. 1B; Fig. S1B), which were mainly related to amino acid metabolism, lipid metabolism, and nucleotide metabolism in *tmexCD1-toprJ1*-positive strains (Fig. 1C; Fig. S1C). In KEGG enrichment analysis, purine metabolism and pyrimidine metabolism were the most severely affected pathways in both *tmexCD1-toprJ1-*carrying *K. pneumoniae* K6 and *E. coli* DH5α (Fig. 1G; Fig. S1G). Specifically, 39 metabolites (10.0%) were enriched in purine metabolism, glycerophospholipid metabolism and pyrimidine metabolism pathways in *K. pneumoniae* K6/pUC19-*tmexCD1-toprJ1*. Meanwhile, 28 metabolites (16.7%) were enriched in purine metabolism, pyrimidine metabolism, glycine, serine, and threonine metabolism, carbon fixation in photosynthetic organisms, and sphingolipid metabolism in *E. coli* DH5α/pUC19-*tmexCD1-toprJ1* (Fig. 1D; Fig. S1D). Unsupervised hierarchical clustering, *Z* scores, and VIP scores were used to rank metabolites whose abundance differed significantly in two strains (Fig. 1D through F ; Fig. S1D through F). A web-based metabolomics pathway analysis tool was used to evaluate the potential impact of differential metabolite abundance on metabolic pathways. Consequently, we identified that these intermediates were important intracellular metabolites of purine metabolism and their downregulation and upregulation perturbed the metabolic pathway (Fig. 1H ; Fig. S1H). These results suggest that the presence and expression of *tmexCD1-toprJ1* indeed alter the metabolic characteristics of bacteria, in which purine metabolism disorder may be closely related to *tmexCD1-toprJ1*-mediated multidrug resistance.

### Differential transcriptomic between *tmexCD1-toprJ1*-negative and -positive bacteria

To gain a deeper understanding of the molecular mechanisms of *tmexCD1-toprJ1*-conferred drug resistance in *K. pneumoniae* K6 or *E. coli* DH5α, we performed transcriptomic analysis of *tmexCD1-toprJ1*-negative and -positive bacteria. PCA plots revealed that significant transcriptomic changes occurred in the *tmexCD1-toprJ1*-positive strains (Fig. S2A and S3A). Compared with the control group, *K. pneumoniae* K6/pUC19-*tmexCD1-toprJ1* showed an upregulation of 984 and a downregulation of 641 differentially expressed genes (DEGs). Meanwhile, *E. coli* DH5α/pUC19-*tmexCD1-toprJ1* led to an upregulation of 866 and a downregulation of 768 DEGs (Fig. S2B and S3B). GO enrichment (Fig. S2C and S3C) and KEGG enrichment analysis were performed to identify the key genes and pathways. The downregulated DEGs were involved in ABC transporters, fructose and mannose metabolism, and pentose and glucuronate interconversions in *K. pneumoniae* K6 (Fig. S2D). And in *E. coli* DH5α, the downregulated DEGs were involved in alanine, aspartate and glutamate metabolism, purine metabolism, pyrimidine metabolism, and two-component system (Fig. S3D). To visualize the pathways, all DEGs annotated in the KEGG database were added to an extensive metabolism map using iPath3 web-based tools (Fig. S2E and S3E). Notably, pyruvate metabolism, purine metabolism, pentose phosphate pathway, glycolysis/gluconeogenesis, two-component system, and citrate cycle (TCA cycle) were the most affected pathway (Fig. S2F through K and S3F through K).

**Fig 2:**
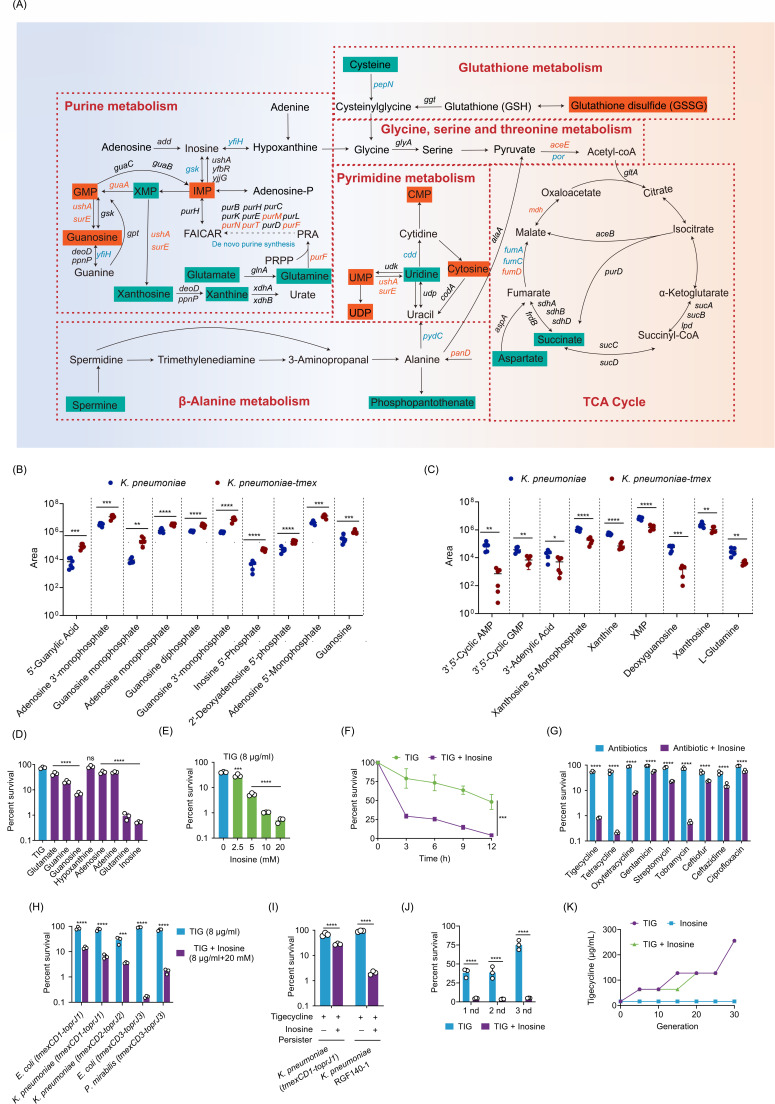
Inosine resensitizes *tmexCD-toprJ-*positive multidrug-resistant bacteria to tigecycline. (**A**) Map of differences metabolic in the purine metabolism between *tmexCD1-toprJ1*-negative and -positive *K. pneumoniae* K6. Upregulated and downregulated metabolites/genes are highlighted in red and green, respectively. (**B**) Scatter plots showing the abundance of downstream purine metabolites in *tmexCD1-toprJ1*-positive *K. pneumoniae* K6. (**C**) Scatter plots showing the abundance of upstream purine metabolites in *tmexCD1-toprJ1*-positive *K. pneumoniae* K6. (**D**) Effect of exogenous purine metabolites on the killing of *K. pneumoniae* RGF 140-1 by tigecycline. (**E**) Percent survival of *K. pneumoniae* RGF 140-1 in the presence of tigecycline and the indicated concentration of glutamine for 12 h. (**F**) Percent survival of *K. pneumoniae* RGF 140-1 in the presence of 20 mM inosine and tigecycline (8 µg/mL) during 12 h. (**G**) Percent survival of *K. pneumoniae* RGF 140-1 in the presence or absence of 20 mM inosine plus various antibiotics for 12 h. (**H**) Percent survival of five *tmexCD-toprJ*-positive tigecycline-resistant bacterial strains in the presence of tigecycline plus 20 mM inosine for 12 h. (**I**) Percent survival of *K. pneumoniae-tmexCD1-toprJ1* and *K. pneumoniae* RGF 140-1 persisters in the presence or absence of tigecycline plus 20 mM inosine for 12 h. (**J**) Percent survival of *K. pneumoniae* RGF 140-1 treated by inosine plus tigecycline after three rounds of consecutive killing. (**K**) MIC of *K. pneumoniae* RGF 140-1 passaged for 30 generations in the presence or absence of inosine (20 mM). Data are means ± SEM from three biological replicates. *P < 0.05; **P < 0.01; ***P < 0.001; ****P < 0.0001.

To explore the role of purine metabolism in *tmexCD1-toprJ1*-mediated drug resistance, we performed an association analysis of the transcriptome and metabolome data to decipher, at a molecular level, the phenotypic differences between *tmexCD1-toprJ1*-negative and -positive bacteria. In particular, the expression of *purF*, *purN*, *purT*, *guaA*, *ushA*, and *surE*, mediating glutamine flux to inosine, GMP, and guanosine were increased, whereas *yfiH* mediating inosine to hypoxanthine was decreased in *K. pneumoniae* K6 (*tmexCD1-toprJ1*) (Fig. 2A). Consistently, the expression of *purF*, *purN*, *purT*, *guaA*, *ushA*, and *surE* was decreased in *E. coli* DH5α (*tmexCD1-toprJ1*) (Fig. S4A). As a consequence, 10 purine metabolism downstream metabolites were significantly upregulated and 9 metabolites were downregulated in the *K. pneumoniae* K6 (*tmexCD1-toprJ1*) (Fig. 2B and C). Furthermore, three purine metabolism downstream metabolites were significantly downregulated and one metabolite was upregulated in the *E. coli* DH5α (*tmexCD1-toprJ1*) (Fig. S4B and S4C). These findings indicate that purine metabolism disorders are closely related to *tmexCD1-toprJ1*-conferred drug resistance.

### Exogenous metabolites resensitize *tmexCD1-toprJ1*-positive bacteria to antibiotics

On the basis of the aforementioned results, we hypothesized that reprogramming purine metabolism might provide a feasible strategy to overcome *tmexCD1-toprJ1*-mediated bacterial resistance. To verify this hypothesis, we tested the effect of exogenous purine metabolism metabolites, including glutamate, guanine, guanosine, hypoxanthine, adenine, glutamine, and inosine, on the survival of a clinical isolate *K. pneumoniae* RGF140-1 (*tmexCD1-toprJ1*) exposed to tigecycline. As a consequence, we found that the addition of seven metabolites except for hypoxanthine drastically enhanced the antibacterial activity of tigecycline (Fig. 2D). Specifically, inosine dose-dependently reduced the survival of drug-resistant bacteria, with 20 mM inosine supplementation leading to a CFU reduction of above 2-log10 compared with TIG alone (Fig. 2E). Also, the synergistic effect of tigecycline and inosine in eliminating *tmexCD1-toprJ1*-positive bacteria was demonstrated in a time-dependent manner (Fig. 2F). Furthermore, we examined the viability of *K. pneumoniae* RGF 140-1 in the different antibiotics, including tetracyclines (tetracycline, oxytetracycline, and tigecycline), aminoglycosides (gentamicin, streptomycin, and tobramycin), cephalosporins (ceftiofur and ceftazidime), and quinolones (ciprofloxacin), with or without inosine. Excitingly, the presence of inosine effectively improved the bactericidal effect of all tested antibiotics against *K. pneumoniae* RGF 140-1 (Fig. 2G). The potentiation of inosine to tigecycline was also evidenced in strains carrying different *tmexCD1-toprJ1* variants (Fig. 2H). We further tested whether exogenous inosine plus tigecycline was also effective against persisters, a small subpopulation of non- or slow-growing bacterial cells that are tolerant to antibiotics killing (22). Consequently, the cell viability of persisters of *K. pneumoniae* K6/pUC19-*tmexCD1-toprJ1* and *K. pneumoniae* RGF140-1 was reduced by 3.6-fold and 40-fold, respectively, by adding inosine to the antibiotic-containing growth medium (Fig. 2I). In addition, it can be found that inosine supplementation contributed to eradicating the mature biofilms of bacteria (Fig. S5A).

To investigate whether the combination of inosine and tigecycline would result in new resistance development, the remaining bacteria, after being killed by inosine plus tigecycline, were subjected to another two rounds of consecutive killing. As shown in Fig. 2J, we found that the remaining bacteria were still susceptible to combinational treatment. Furthermore, *K. pneumoniae* RGF140-1 was serially propagated in gradually increasing concentrations of tigecycline in the absence or presence of inosine. After being passaged for 30 generations, bacteria had the same value of MICs regardless of the absence or presence of inosine (Fig. 2K). These data together suggest that the addition of purine metabolism metabolites, especially inosine, effectively enhances antibiotic activity against *tmexCD1-toprJ1* -positive bacteria without detectable resistance development.

### Inosine promotes the uptake of tigecycline by upregulating bacterial PMF

**Fig 3:**
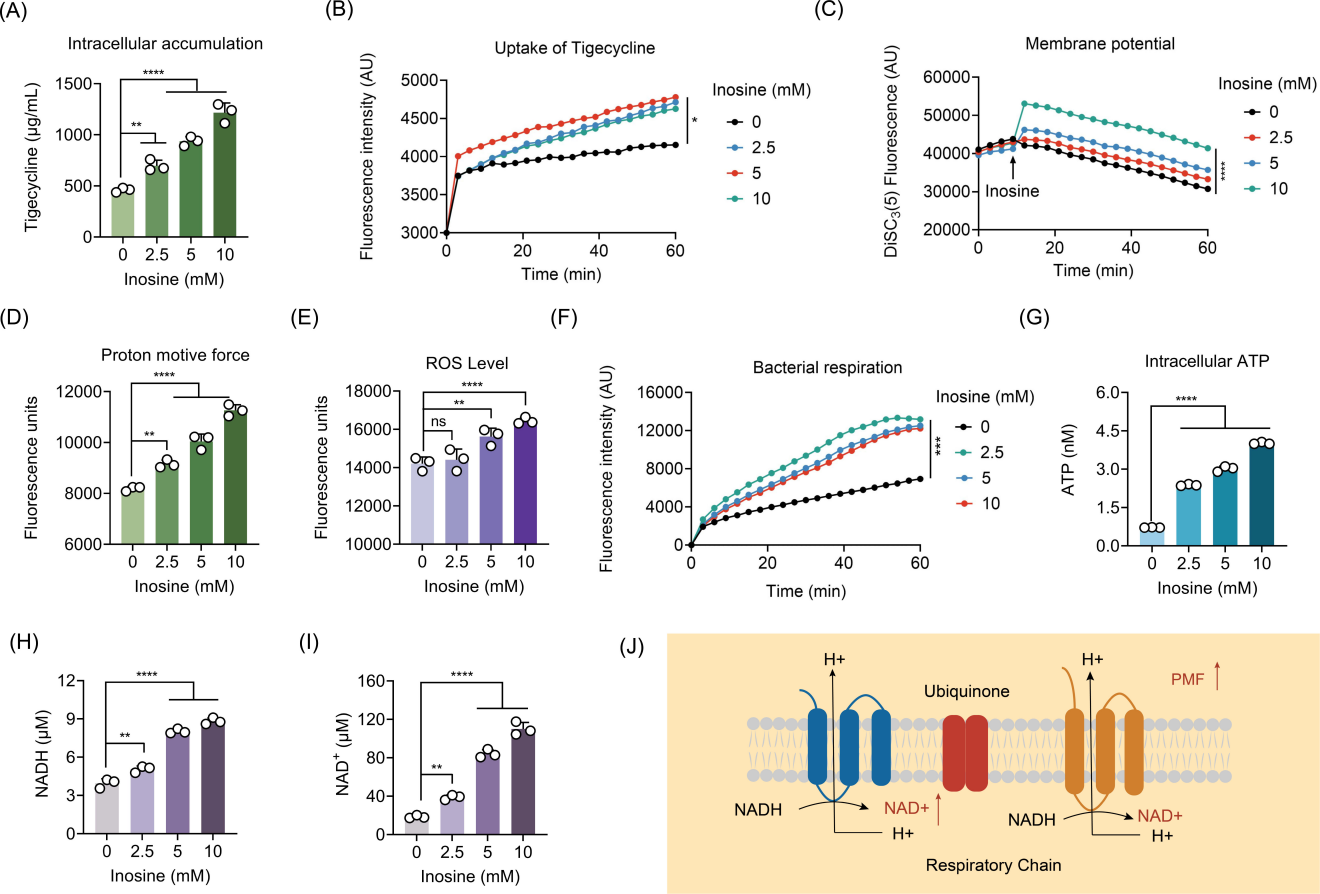
Inosine enhances the uptake of tigecycline by upregulating bacterial PMF and energy metabolism. (**A**) The intracellular accumulation of tigecycline in *K. pneumoniae* 140-1 after treatment with increasing concentrations of inosine by LC-MS/MS analysis. Initial concentration of tigecycline was 16 µg/mL in *K. pneumoniae* 140-1. (**B**) Effect of inosine on the uptake of tigecycline in *K. pneumoniae* 140-1. (**C**) Inosine dissipates membrane potential in bacteria. Fluorescence intensity of DiSC_3_(5) in *K. pneumoniae* RGF 140-1 after treatment with increasing concentrations of inosine was monitored. Drugs were added into DiSC3(5)-probed cells at 60 min. (**D**) Upregulation of ∆pH in BCECF-AM-labeled *K. pneumoniae* RGF 140-1 cells after exposure to varying concentrations of inosine. (**E**) The generation of ROS in *K. pneumoniae* RGF 140-1 treated by inosine. (**F**) Respiration of *K. pneumoniae* RGF 140-1 cultures in the logarithmic growth phase in resazurin assay. (**G**) Intracellular ATP level in *K. pneumoniae* RGF 140-1 after exposure to inosine. ATP level was determined based on bioluminescent reaction catalyzed by firefly luciferase. (**H and I**) Intracellular NADH and NAD^+^ concentration in *K. pneumoniae* 140-1 in the presence of inosine. (**J**) Schematic diagram of mechanism by which inosine regulates PMF. Data are means ± SEM from three biological replicates. *P < 0.05; **P < 0.01; ***P < 0.001; ****P < 0.0001.

Having shown that inosine drastically potentiated tigecycline activity against resistant pathogens, we next sought to elucidate the underlying mechanisms. Considering that the antibacterial activity of tigecycline is dependent on the inhibition of protein synthesis (23), thus intracellular accumulation of tigecycline is essential for its activity. As expected, a dose-dependent accumulation of tigecycline in *K. pneumoniae* RGF140-1 by inosine was observed through LC-MS/MS analysis (Fig. 3A). Given that the intracellular accumulation is the combined result of the uptake and efflux of drugs, thus we further measured the function of the uptake and efflux pump in bacteria under the treatment of inosine. As expected, inosine supplementation remarkably enhanced the uptake of tigecycline in *K. pneumoniae* RGF140-1 (Fig. 3B). Furthermore, inosine modestly inhibited efflux pump function (Fig. S5B). The results indicated that the action of inosine was highly dependent on the increased tigecycline uptake in *tmexCD1-toprJ1* -positive bacteria. It has been shown that bacterial PMF and porin expression are essential for the uptake of drug (24, 25). Notably, the PMF is subsequently necessary for ATP synthesis by the F1F0-ATPase and for the transport of various solutes (26). Generally, PMF is made up of the sum of two parameters: the electric potential (ΔΨ) and the transmembrane proton gradient (ΔpH) (27). It has been indicated that the uptake of tetracyclines by bacterial cells depends on ΔpH, whereas aminoglycosides utilize the ΔΨ component for transport. Therefore, we hypothesized that inosine might target the ΔΨ component of PMF. To test our hypothesis, a fluorescent probe 3,3′-dipropylthiadicarbocyanine iodide [DiSC_3_(5)] (28) was used to assess membrane potential changes induced by inosine in *K. pneumoniae* RGF140-1. As expected, the addition of inosine significantly increased the fluorescence (Fig. 3C). The increased fluorescence indicated the release of DiSC_3_(5) from the cytoplasmic membrane to the extracellular milieu due to the disruption of ∆ψ. To maintain a constant value of PMF, the dissipation of ∆ψ would be compensated by the increasing ΔpH. To test whether ΔpH will be compensatory upregulated, a membrane-permeable fluorescent probe termed BCECF-AM (29) was used to monitor intracellular pH changes in *K. pneumoniae* RGF140-1. Interestingly, inosine led to increased ΔpH in *K. pneumoniae* RGF140-1 (Fig. 3D). These data were in agreement with the previous observations that the increasing ΔpH contributes to the uptake of tigecycline. Bacterial PMF is related to the production of reactive oxygen species (ROS) and ATP (30–32). Therefore, we further determined the ROS generation in *K. pneumoniae* RGF140-1 under exposure of increasing inosine using DCFH-DA (33). Surprisingly, inosine dose-dependently markedly promoted the generation of ROS (Fig. 3E), which has been recognized as one of the common mechanisms in the antibiotic-mediated killing of bacteria (31). The overproduction of ROS gives an interpretation of synergistic bactericidal activity of inosine and tigecycline combination.

### Inosine upregulates the TCA cycle and enhances the contents of succinate

In addition to the PMF, the activation of TCA cycle can also increase the production of ATP, respiration, NADH, and stimulate the uptake of antibiotics (15). Therefore, we set out to investigate the effect of inosine on the bacterial TCA cycle and bacterial respiration. As expected, we found that stationary-phase *K. pneumoniae* RGF140-1 displayed a weak respiration rate, whereas the inosine supplementation remarkedly enhanced bacterial respiration and ATP level (Fig. 3F and G). These results indicated that the addition of inosine stimulated bacterial respiration and converted resistant cells to metabolically active cells. In addition, the intracellular NADH and NAD^+^ levels in resistant bacteria were measured after incubation with inosine. The levels of NADH and NAD^+^ were both dose-dependently upregulated in the inosine-treated bacteria (Fig. 3H and I). These results indicated that exogenous inosine enhanced NAD^+^ levels, in turn, stimulated H^+^ from intracellular to extracellular, ultimately leading to an increase in PMF. This supports our previous findings that ΔpH increases in the presence of inosine (Fig. 3J).

**Fig 4:**
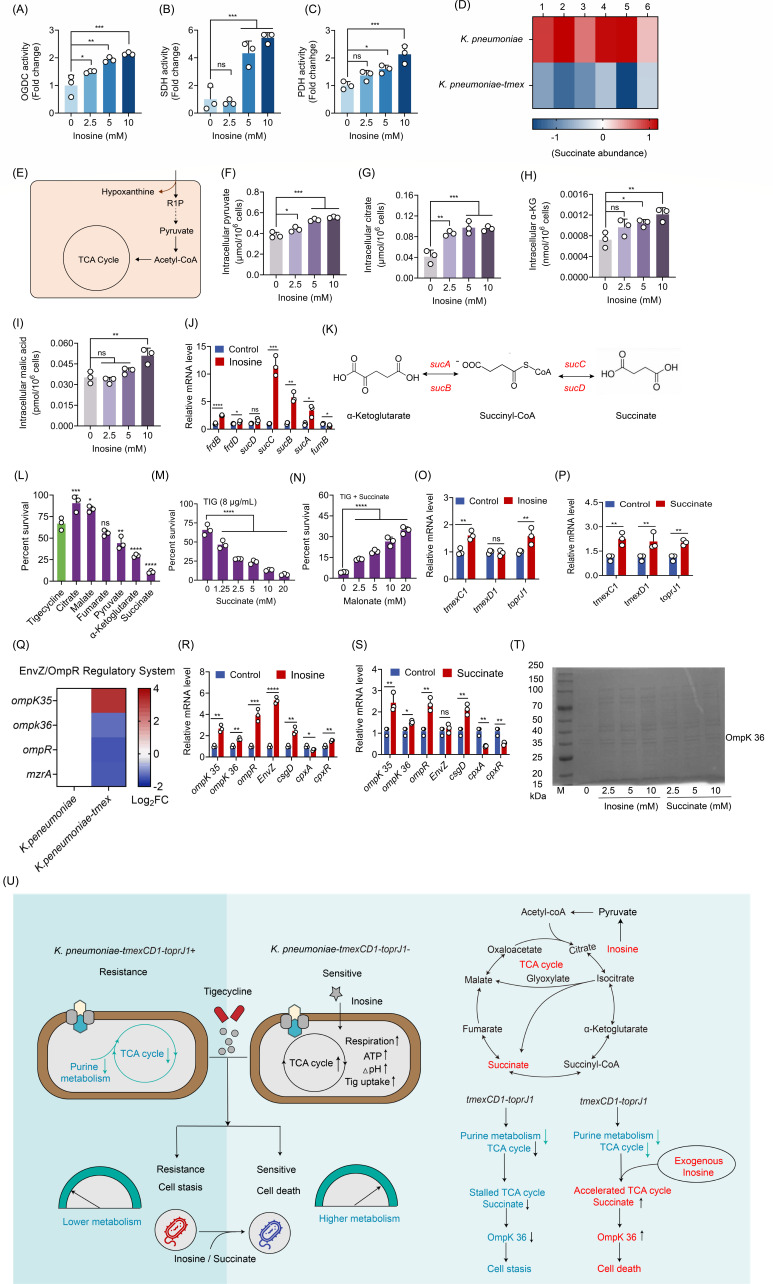
Inosine promotes succinate biosynthesis to potentiate tigecycline activity. (**A–C**) Activity of three OGDC, SDH, and PDH enzymes of the TCA cycle in the presence or absence of exogenous inosine. (**D**) Scatter plots showing the abundance of succinate in *tmexCD1-toprJ1*-negative and -positive bacteria. (**E**) Superimposed on metabolic pathways related to inosine and the TCA cycle. (**F–I**) The intracellular accumulation of pyruvate (**F**), citrate (**G**), α-ketoglutarate (**H**), and malate (**I**) in *tmexCD1-toprJ1-*positive bacteria exposed to increasing concentrations of inosine. (**J**) Quantification of the transcriptional level of TCA cycle-related genes in *K. pneumoniae* RGF 140-1 treated with or without inosine. (**K**) Metabolic pathway of α-ketoglutarate to succinate. (**L**) Effect of exogenous TCA cycle metabolites on the killing of *K. pneumoniae* RGF 140-1 by tigecycline. (**M**) Percent survival of *K. pneumoniae* RGF 140-1 in the presence of tigecycline plus inosine and the increasing concentrations of malonate. (**N**) Percent survival of *K. pneumoniae* RGF 140-1 in the presence of tigecycline and the indicated concentration of succinate for 24 h. (**O and P**) Quantification of the transcriptional level of *tmexC1*, *tmexD1*, and *toprJ1* genes in *K. pneumoniae* RGF 140-1 treated with or without inosine (**O**) or succinate (**P**). (**Q**) Selected differential expression genes involved in the EnvZ/OmpR regulatory system. Color shades indicate Log_2_ fold changes of increased (red) or decreased (blue) gene expression in *tmexCD1-toprJ1*-positive *K. pneumoniae*. (**R and S**) Quantification of the transcriptional level of *ompK 35*, *ompK 36*, *ompR*, *csgD*, *EnvZ*, *cpxA*, and *cpxR* genes in *K. pneumoniae* RGF 140-1 treated with or without inosine (**R**) or succinate (**S**). (**T**) Sodium dodecyl sulfate-polyacrylamide gel electrophoresis (SDS-PAGE) analysis of outer membrane porins (OMPs) from *K. pneumoniae* RGF 140-1 strains treated by inosine or succinate. (**U**) Proposed mechanisms for cell killing in the presence of inosine plus tigecycline. Data are means ± SEM from three biological replicates. *P < 0.05; **P < 0.01; ***P < 0.001; ****P < 0.0001.

Further enzymatic activity assays indicated that exogenous inosine enhanced the activity of α-ketoglutarate dehydrogenase (OGDC), succinate dehydrogenase (SDH), and pyruvate dehydrogenase (PDH) (Fig. 4A through C), suggesting that the TCA cycle was readjusted by inosine. Given that inosine strongly activated the TCA cycle, we speculated that the content of TCA cycle intermediates may alter correspondingly. Interestingly, we found that succinate abundance was suppressed to a greater extent in *K. pneumoniae* K6/pUC19-*tmexCD1-toprJ1* by LC-MS/MS-based metabolomics (Fig. 4D). It has been shown that the ribose subunit of inosine can enter into central metabolic pathways to provide ATP and biosynthetic precursors (34) (Fig. 4E). Furthermore, the intracellular pyruvate, citrate, α-KG, and malic acid content in *tmexCD1-toprJ1-*carrying *K. pneumoniae* in the presence and absence of exogenous inosine were measured. As a result, exogenous inosine enhanced the TCA cycle intermediate pyruvate, citrate, α-KG, and malic acid content (Fig. 4F through I). Consistently, we also observed increased transcription of the TCA cycle-related genes in *K. pneumoniae* RGF140-1 treated with inosine in RT-qPCR analysis (Fig. 4J). In addition, exogenous inosine significantly enhanced the expression of succinate biosynthesis-related genes (Fig. 4K). The susceptibility of resistant bacteria to tigecycline was restored potently by pyruvic acid, α-ketoglutaric acid, and succinate (Fig. 4L). These results suggested that carbon metabolism plays an essential role in bacterial susceptibility to tigecycline. Consistently, the addition of a TCA cycle inhibitor, malonate, compromised the antibacterial activity of tigecycline (Fig. 4M). Also, exogenous succinate elevated the sensitivity of *K. pneumoniae* RGF 140-1 to tigecycline in a dose-dependent manner (Fig. 4N). These results support the conclusion that exogenous inosine crosses the bacterial membrane and ultimately enters into the intracellular TCA cycle pathway, leading to the accumulation of succinate.

### The mechanisms by which succinate restores bacterial susceptibility to antibiotics

To investigate the underlying mechanisms of succinate-enabled tigecycline killing, we quantified the transcription of *tmexCD1-toprJ1* gene in *K. pneumoniae* RGF140-1. However, we found that both succinate and inosine had minimal effects on the expression of *tmexC1*, *tmexD1*, and *toprJ1* genes (Fig. 4O and P), indicating that the potentiation of inosine and succinate to tigecycline was not related to the expression changes of *tmexCD1-toprJ1*. Considering the important roles of porin expression in antibiotic influx, we speculated that succinate and inosine may act through the regulation of porins. Through the induction of *mzrA* gene, *CpxR* indirectly activates the histidine kinase EnvZ, which is part of the EnvZ/OmpR two-component system (35). EnvZ/OmpR two-component system influences membrane permeability through the regulation of porins, such as OmpK 35 and OmpK 36. Thus, we reasoned that succinate may hijack the EnvZ/OmpR system to allow antibiotic influx through OmpK, a well-recognized porin for antibiotics (34). To test this hypothesis, we first analyzed the EnvZ/OmpR two-component system-related gene expression level in the presence of *tmexCD1-toprJ1*. Specifically, *mzrA*, *OmpR*, and *OmpK 36* genes in *tmexCD1-toprJ1-*bacteria were significantly downregulated (Fig. 4Q). Conversely, an increased transcription of *ompK 35*, *ompK 36*, *ompR*, *EnvZ*, *csgD*, *cpxA*, and *cpxR* in *K. pneumoniae-tmexCD1-toprJ1* treated with succinate or inosine was found (Fig. 4R and S). These data suggest that succinate activated EnvZ, which modulated the intracellular level of phosphorylated OmpR. Under a higher level of OmpR-P, transcription of OmpK 36 is activated, as described previously (36–38). To verify it, we detected the expression of the outer membrane protein OmpK 36 by SDS-PAGE. Consequently, we found that the addition of succinate or inosine enhanced the expression of OmpK 36 (Fig. 4T). Collectively, these data suggest that inosine supplementation-induced metabolic reprogramming effectively restores the susceptibility of *tmexCD1-toprJ1*-positive bacteria to tigecycline by upregulating PMF and porin expression to promote antibiotic uptake (Fig. 4U).

### Inosine reverses *tmexCD1-toprJ1*-mediated tigecycline resistance *in vivo*

**Fig 5:**
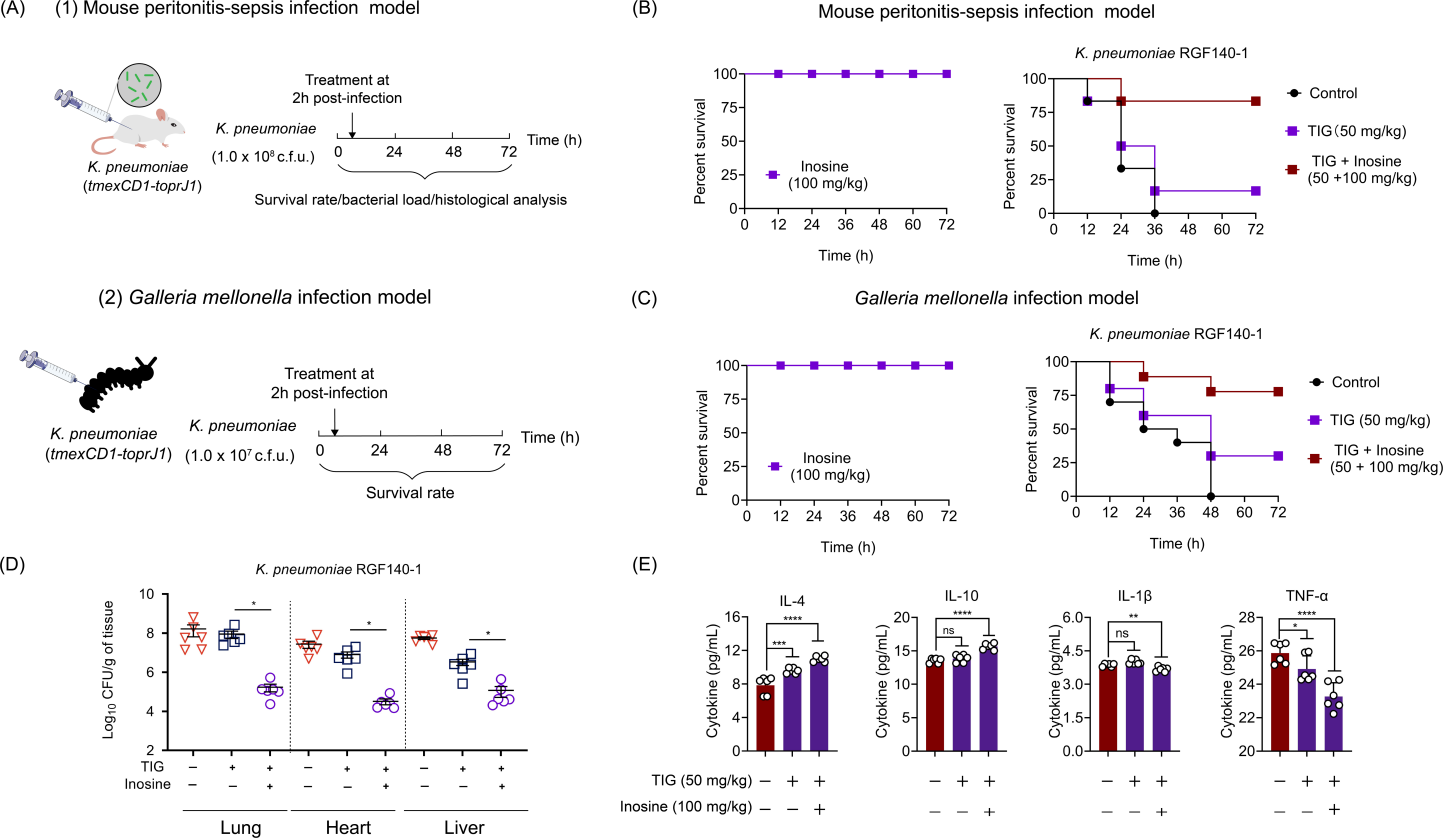
*In vivo* therapeutic effect of inosine plus tigecycline against *tmexCD-toprJ*-carrying *K. pneumoniae*. (**A**) Scheme of the experimental protocols for mouse peritonitis-sepsis infection model and *Galleria mellonella* infection model. In the infection models, mice (*n* = 6 per group) and larvae (*n* = 10 per group) were infected by *K. pneumoniae* RGF 140-1 strains (*tmexCD1-toprJ1*), respectively, and treated with a single dose of tigecycline (50 mg/kg) and a combination of tigecycline plus inosine (50 + 100 mg/kg) at 2 h post-infection. (**B and C**) Survival rate of infected larvae (**B**) and mice (**C**) after tigecycline or combination treatment for 3 days. The *in vivo* safety of inosine (100 mg/kg) in two animals was also evaluated. (**D**) The lung, heart, and liver bacterial loads in single and combination therapy at 2 days post-infection. (**E**) The levels of inflammatory factors (IL-4, IL-1β, IL-10, and TNF-α) in the infected mice after treatment with tigecycline alone or in combination with inosine. Data are presented as means ± SEM. *P < 0.05; **P < 0.01; ***P < 0.001; ****P < 0.0001. ns, not significant.

Given the effective restoration of tigecycline activity *in vitro* by inosine, we next sought to explore the potential of inosine to rescue tigecycline effectiveness *in vivo*. To achieve this, we evaluated the *in vivo* efficacy of tigecycline alone and in combination with inosine using two animal infection models (Fig. 5A). In the mouse peritonitis model, we found that mice infected with *K. pneumoniae* RGF 140-1, a *tmexCD1-toprJ1* positive strain, all died during 3 days. And the tigecycline monotreatment achieved a survival rate of 16.7%. By contrast, the combination of inosine and tigecycline dramatically increased the survival of mice to 83% (Fig. 5B). Also, we demonstrated the in *vivo* safety of inosine by daily intraperitoneal injection in mice, using a dose of 100 mg/kg (Fig. 5B). This survival advantage under combination treatment was also validated in a *Galleria mellonella* infection model (Fig. 5C)**.** Correspondingly, the colony-forming units (CFUs) in the lungs, hearts, and livers of mice were reduced in the combination treatment group (Fig. 5D). Meanwhile, the anti-inflammatory factors contents of IL-4 and IL-10 in the lungs of infected mice were significantly increased under combination therapy (*P* < 0.05). By contrast, the pro-inflammatory factors TNF-α and Il-1β in the lungs of infected mice were significantly reduced in the combination therapy group (Fig. 5E). The pathological damage in infected mice in different groups was evaluated by histopathological analyses. Under inosine and tigecycline combination therapy, the pathological damages were effectively alleviated (Fig. S6). These data together demonstrate the *in vivo* effectiveness of tigecycline in combination with inosine against *tmexCD1-toprJ1-*positive bacterial infections.

## DISCUSSION

Tigecycline is one of the few antibiotics active against MDR Gram-negative bacteria (39, 40). However, the acquisition of *tmexCD-toprJ* in clinically relevant pathogens from human and animal sources confers high levels of tigecycline resistance (10). Therefore, developing novel strategies to rescue tigecycline efficacy is urgently warranted. Traditionally, combinational approaches, including the combination of tigecycline and other antibiotics/non-antibiotic drugs (41), have been explored to tackle tigecycline resistance. For example, FDA-approved drugs, such as melatonin (42), benzydamine (43), and metformin (44), have demonstrated the ability to restore the antibacterial activity of tigecycline. However, concerns also arise when additional drugs are used, as this may lead to the emergence of new drug resistance. For example, inhibitors like sulbactam, which inhibit extended-spectrum β-lactamase, can induce resistance in various bacteria (45, 46). As new resistance pathways emerge, there is a need for effective therapies with unique mechanisms to overcome AMR. Alternatively, pharmacological strategies targeting metabolic reprogramming have shown increasing promise, bypassing the need to discover or design new drugs or scaffolds. In this study, we identified that purine metabolism disorder was closely linked to *tmexCD-toprJ*-mediated multidrug resistance in Gram-negative bacteria. Consistently, previous research has shown that antibiotic stress modulated bacterial purine metabolism (47). Therefore, the reconstruction of purine pool by targeting purine metabolism offers a promising and distinct strategy for bacterial killing (48). Most importantly, we found that inosine supplementation, a key purine metabolite, effectively restored the susceptibility of *tmexCD-toprJ-*positive bacteria to multiple antibiotics, particularly for tigecycline. Noteworthy, although the abundance of inosine had been reported to be repressed in multidrug-resistant uropathogenic bacteria (25), its potential in reversing *tmexCD-toprJ-*positive bacteria remains unknown. Our mechanistic studies demonstrated that the enhanced uptake of tigecycline was the primary mechanism of action of inosine to overcome *tmexCD-toprJ-*mediated multidrug resistance. On the one hand, inosine was evidenced to upregulate the ΔpH component of bacterial PMF, which was critical for the uptake of tetracyclines (49). Besides, we also found that inosine accelerated the TCA cycle and succinate biosynthesis, leading to the accumulation of succinate. Interestingly, we found that succinate had no impact on the expression of *tmexCD1-toprJ1* resistance gene, but significantly enhanced the expression of OmpK 36, which is a downstream porin protein that plays a crucial role in antibiotic uptake through the inosine-succinate-EnvZ/OmpR-OmpK 36 pathway. Multiple lines of evidence indicated that OmpK 36 is a nonspecific antibiotic porin, and its deletion results in a high level of resistance to various types of antibiotics, including β-lactams, aminoglycosides, fluoroquinolones, and tetracyclines in *K. pneumoniae* (50–52). In agreement with our findings, several previous studies have also demonstrated that modulating the metabolism of antibiotic-resistant bacteria and stimulating the transport of extracellular antibiotics through the bacterial membrane into the intracellular environment contribute to improving antibiotic activity (15, 25).

Recent studies demonstrated that inosine has neuroprotective, cardioprotective, and immunomodulatory effects (53). The combination of inosine and tigecycline could potentially be explored in clinics to treat infections by multidrug-resistant bacteria. With regard to the safety of inosine, the doses of inosine used in this study were lower than previously reported (54, 55). Consistent with our findings, it has been shown that rats receiving 100 mg/kg inosine were still safe (56, 57). Additionally, inosine supplementation via oral administration or intravenous infusion has been shown to be safe and tolerable in recent clinical trials (58). The highest dose of tigecycline used in this study *in vivo* was 50 mg/kg. At this dose, we did not observe any aberrant behavior from the animal studies. At this dose combination, inosine plus tigecycline achieved great therapeutic outcomes in multiple animal models of infection.

In conclusion, our data suggest that *tmexCD-toprJ*-negative and -positive bacteria display distinct metabolic flow and metabolic characteristics, especially in purine metabolism. Furthermore, we provide a combinational approach of inosine or succinate with tigecycline, which could potentially tackle *tmexCD-toprJ*-positive bacteria both *in vitro* and in multiple animal infection models. Compared with conventional antibacterial strategies, this approach eliminates the need to develop new drugs and reduces the concern of acquiring resistance to future antibiotics, thereby providing novel insights into the treatment of recalcitrant infections by drug-resistant pathogens.

## MATERIALS AND METHODS

### Bacterial strains and chemical agents

All strains used in this study were listed in Table S1 and S2. *E*. *coli* DH5a and *K. pneumoniae* K6 (ATCC 700603), purchased from Fuxiang Biological Company (Shanghai, China), were utilized as standard strains in this study. All antibiotics were obtained from Shanghai yuanye Bio-Technology and other chemicals were purchased from Solarbio (Beijing, China).

### Plasmids and genetic manipulations

DNA fragments containing *tmexCD1-toprJ1* were amplified using primers listed in Table S3 in the supplemental material and ligated into pUC19, yielding pUC19-*tmexCD1-toprJ1*. The recombinant plasmid pUC19-*tmexCD1-toprJ1* and pUC19 plasmid were transformed into reference strains *K. pneumoniae* K6/*E. coli* DH5α, respectively.

### Metabolomic and data analysis

Sample preparation and LC-MS/MS analysis were carried out as previously described (59). Briefly, logarithmic phase cells were collected and suspended in 1 mL of pre-cooled methanol for the extraction of cellular metabolites. The sample was sonicated at a low temperature (30 min/once, twice) and then centrifuged for 20 min (14,000 × *g*, 4°C). The supernatant was dried in a vacuum centrifuge. For LC-MS analysis, the dried samples were dissolved in 100 µL acetonitrile/water (1:1, vol/vol), adequately vortexed and then centrifuged (14,000 rpm, 4°C, 15 min). The supernatants were collected for the LC-MS/MS analysis. Analyses were performed using a UHPLC (1290 Infinity LC, Agilent Technologies) coupled to a QTRAP (AB Sciex 5500). The pretreatment of LC/MS raw data were performed by Progenesis QI (Waters Corporation, Milford, USA) software. At the same time, the metabolites were identified by searching databases, and the main database was the HMDB. Then, the R package “ropls” (Version 1.6.2) was used to perform PCA and orthogonal least partial squares discriminant analysis (OPLS-DA). The metabolites with VIP >1, *P* < 0.05 were determined as significantly different metabolites based on the Variable importance in the projection (VIP) obtained by the OPLS-DA model. Differential metabolites among the two groups were mapped into their biochemical pathways through metabolic enrichment and pathway analysis based on the KEGG database. Enrichment analysis was used to analyze a group of metabolites in a function node.

### Transcriptomic analysis

Briefly, the total RNA of logarithmic phase bacteria was extracted and quantified according to the ratio of OD260/280 nm using a Nanodrop spectrophotometer (Thermo Fisher Scientific), and rRNA was depleted using the Ribo-off rRNA depletion kit (Vazyme). Subsequently, sequenced on Hiseq2000 with Truseq SBS Kit v3‐HS (200 cycles) (Illumina) with the read length as 2 × 100 (PE100). Raw sequencing reads were filtrated and mapped against the reference genome of *K. pneumoniae* K6/*E. coli* K12. The FPKM (Fragments Per Kilobase of transcript per Million mapped reads) method was used to identify differentially expressed genes with *P* values ≤0.05 and fold change (FC) values ≥2 (log2 FC ≥1 or log2 FC ≤−1). Functional annotation was performed by comparing with GO and KEGG databases. RSEM software was used to quantitatively analyze the expression levels of genes, and DESeq2 was used to identify DEGs. Three independent experimental replicates were used for transcriptomic analysis.

### MIC analysis

MIC was measured using the standard broth microdilution method. All antibiotics were twofold diluted in MHB and equally mixed with bacterial suspensions in a 96-well microtiter plate (Corning, New York, USA). MIC values were defined as the lowest concentrations of drugs with no visible growth of bacteria after 18 h incubation at 37°C.

### Measurement of bacterial survival

A single colony was inoculated into 30 mL LB medium in 50 mL flasks and grown for 16 h at 37°C. Unless otherwise stated, samples were collected by centrifugation at 8,000 × *g* for 5 min, washed with 10 mL 0.9% saline and re-suspended in the modified M9 medium to 10^7^ CFU/mL. Subsequently, metabolites and an appropriate amount of antibiotics were added as required. After 12 h, 100 µL aliquots of samples were removed. The aliquots of samples were serially diluted and CFU/mL was determined by plating method. Experiments were performed with three biological replicates.

### Time-dependent killing studies

Overnight cultures of *K. pneumoniae* RGF140-1 (carrying *tmexCD1-toprJ1*) were diluted 1/1,000 into MH broth at 37°C for 4 h. The cells were treated with inosine or TIG alone, or their combination for 3, 6, 9, and 12 h. Finally, suspensions were plated on LB medium and incubated overnight at 37°C. Bacterial numbers were calculated. LB broth with no drugs was used as a control. Experiments were performed with three biological replicates.

### Persister assays

Bacteria were cultured in MHB overnight to stationary phase with shaking at 37°C, 200 rpm. Then, the overnight culture (6 × 10^9^ CFU/mL cells) was treated with 120 µg/mL tigecycline for 16 h to kill growing bacteria and enrich persisters as described previously (25). Persisters were treated with inosine or TIG alone or their combination for 12 h. To determine bacterial counts, 100 µL of cultures was removed and then serially diluted. Percent survival was determined by dividing the CFU obtained from a treated sample by the CFU obtained from the control.

### Resistance development assessment

Overnight cultures of *K. pneumoniae* RGF140-1 were diluted 1/1,000 into MH broth and incubated for 4 h at 37°C with sharking at 200 rpm. Bacteria were treated with TIG (16 µg/mL), inosine (10 mM), or their combination. After incubation at 37°C for 12 h, the MIC of the cultures was determined. Meanwhile, a 1/1,000 dilution of the bacteria culture was performed into a fresh medium supplemented with 0.5 × MIC of TIG for the next passages. This experiment was performed for 30 passages, and the MIC increase of TIG was calculated. The remaining bacteria were subjected to another two rounds of consecutive killing.

### Proton motive force assay

Proton motive force assay was carried out as previously described (60). Briefly, the proton motive force assay of *K. pneumoniae* RGF140-1 treated by inosine was measured with pH-sensitive fluorescence probe BCECF-AM (20 × 10^−6^ M). After the fluorescence stabilized, varying inosine was added. For all BCECF-AM experiments, the excitation and emission wavelengths on the fluorescence spectrometer were set to 500 and 522 nm, respectively.

### Membrane depolarization assay

Membrane depolarization assay was performed as previously described (60). In brief, logarithmic phase *K. pneumoniae* RGF140-1 was washed with M9 to an OD_600_ of 0.5 and incubated with DiSC_3_(5) (0.5 × 10^−6^ M) for 30 min. Finally, varying concentrations of inosine were added to the 190 µL of DiSC_3_(5)-loaded cells. For membrane depolarization experiments, the excitation wavelength was 622 nm, and the emission wavelength was 670 nm, with an interval of 3 min for 60 min.

### Total ROS measurement

The levels of ROS in *K. pneumoniae* RGF140-1 treated by inosine were measured with 2′,7′-dichlorodihydrofluorescein diacetate (DCFH-DA), following the manufacturer’s instruction (Beyotime, Shanghai, China). After incubation for 1 h, the fluorescence intensity was immediately measured with the excitation wavelength at 488 nm and emission wavelength at 525 nm.

### ATP determination

Intracellular ATP levels of *K. pneumoniae* RGF140-1 were determined using an Enhanced ATP Assay Kit as previously described (61). The total ATP levels in samples were calculated based on the standard curve of luminescence signals versus concentrations of ATP standard solution.

### Uptake of tigecycline

Tigecycline uptake was evaluated by monitoring the fluorescence change of the TIG in bacteria as previously described (62). *K. pneumoniae* RGF140-1 was grown to an OD_600_ of 0.5. The cells were centrifuged at 3,500 rpm for 10 min and washed in an equal volume of PBS three times. Subsequently, tigecycline at MIC alone or with various concentrations of inosine was added to the 96-well plates containing cell suspensions at 100 µL/well. An infinite Microplate reader was used to monitor the fluorescence intensity with an excitation wavelength of 405 nm and emission wavelength of 535 nm.

### Tigecycline accumulation analysis

The logarithmic phase *K. pneumoniae* RGF140-1 was washed with M9 to an OD_600_ of 0.5, and then cells were pelleted by centrifuging at 12,000 × *g* for 10 min and diluted into 10^10^ CFUs/mL by M9. The suspension was aliquoted into 1.5 mL tubes. Then, TIG at the MIC concentration together with varying concentrations of inosine was added and bacteria were cultured at 37°C with shaking at 200 rpm. After 15 min, cultures were centrifuged at 12,000 × *g* for 3 min for the following drug extraction, according to our previous study (43). Tigecycline accumulation was detected by an Agilent 1260 Infinity HPLC system combined with an AB SCIEX QTRAP 6500 mass spectrometer (ABSciex, Foster City, CA, USA).

### Bacterial respiration assay

The logarithmic phase *K. pneumoniae* RGF140-1 was washed with M9 to an OD_600_ of 0.5. Resazurin (63) was added to the bacteria culture with a final concentration of 0.1 µg/mL, followed by varying concentrations of inosine. The cells were aliquoted into a 96-well plate, the fluorescence units were immediately measured with the excitation wavelength at 550 nm and emission wavelength at 590 nm for 30 min.

### NAD^+^/NADH determination

NAD^+^/NADH determination was performed based on a previous study (64). In brief, logarithmic phase *K. pneumoniae* RGF140-1 was washed with M9 broth to an OD_600_ of 0.5. After being treated with various concentrations of inosine for 6 h, the cells were washed and resuspended with 200 µL precooled extraction buffer. The lysate was centrifuged at 12,000 × *g* for 10 min at 4°C and the supernatant was divided into two parts; one was used for the determination of the total amount of NAD^+^/NADH, and the other was used for detecting the amount of NADH only.

### Measurement of enzyme activity

SDH, PDH, and OGDH were measured by succinate dehydrogenase, pyruvate dehydrogenase, and a-ketoglutarate dehydrogenase Activity Assay Kit (Beijing Solarbio Science & Technology Co., Ltd). In brief, logarithmic phase *K. pneumoniae* RGF140-1 was washed with M9 broth to an OD_600_ of 0.5. After being treated with various concentrations of inosine for 6 h, the cells were disrupted by sonic oscillation.

### Determination of organic acid content in TCA cycle

Pyruvate, citrate, α-ketoglutarate, and malic acid concentrations were detected as previously reported (65). In brief, logarithmic phase *K. pneumoniae* RGF140-1 was washed with M9 broth to an OD_600_ of 0.5. After treatment with various concentrations of inosine for 6 h, aliquots of 1 mL samples were sonicated for 3 min, and the resulting supernatant was used for the detection of pyruvate, citrate, α-ketoglutarate, and malic acid.

### RT-PCR analysis

*K. pneumoniae* RGF140-1 was grown overnight in LB broth and diluted 1/100 into 1 mL fresh LB supplemented with inosine. After bacterial cells were grown to the mid-log phase at 37°C, total RNA was extracted using Bacteria RNA Extraction Kit (Vazyme). The extracted RNA was reverse-transcribed using PrimeScript RT Kit and gDNA Eraser (Takara, Dalian, China) following the instructions. RT-qPCR analysis was performed by 7500 Fast Real-Time PCR System (Applied Biosystem, CA, USA) using the ChamQ SYBR Color qPCR Master Mix (Vazyme) with the primers (Table S2). Thermal cycling was performed by a two-step PCR amplification standard procedure, 40 cycles of 95°C for 30 s, 95°C for 10 s, and 60°C for 30 s. 2^−ΔΔCt^ method was applied to calculate the fold change of mRNA expression relative to a reference gene (16S rRNA).

### Outer membrane protein analysis

*K. pneumoniae* RGF140-1 was grown overnight in LB broth and diluted 1/100 into 1 mL fresh LB supplemented with inosine or succinate. After incubation for 6 h at 37°C with 200 rpm, bacterial cells were collected, washed, resuspended in M9 broth, and disrupted by sonic oscillation. BCA Protein Assay Kit assay was used to detect protein concentrations and adjust the protein concentration of each sample to be consistent. Bacterial cells were directly lysed in 5 × SDS loading buffer and boiled for 10 min. After centrifugation, 10 µL of total protein extracts were separated by 12% sodium dodecyl sulfate-polyacrylamide gel electrophoresis (SDS-PAGE).

### Animal studies

Six- to eight-week-old female BALB/c mice (18–20 g) were obtained from the Comparative Medicine Centre of Yangzhou University (Jiangsu, China). All experiments were performed under the guidelines of Jiangsu Laboratory Animal Welfare and Ethical of Jiangsu Administrative Committee of Laboratory Animals (permission number, SYXK-2022-0044). The laboratory animal usage license number is SCXK-2022-0009, certified by the Jiangsu Association for Science and Technology.

### Mouse peritonitis infection model

Female BALB/C mice (*n* = 6 per group) were intraperitoneally infected with a lethal dose of 1.0 × 10^8^ CFU *K. pneumoniae* RGF140-1 suspension. After 2 h post-infection, mice were treated with a single dose of tigecycline (50 mg/kg) and a combination of tigecycline plus inosine (50 + 100 mg/kg) via intraperitoneal injection. Survival rates of treated mice were recorded for 3 days. The lung was aseptically removed and divided into three parts for CFU determination, cytokines measurement, and HE staining.

### *Galleria mellonella* infection model

*G. mellonella* larvae were randomly divided into three groups (*n* = 10 per group) and infected with 1.0 × 10^7^ CFU *K. pneumoniae* RGF140-1 (10 µL, 1.0 × 10^7^ CFU per larvae) at the right posterior gastropoda. At 2 h post-infection, larvae were injected with a single dose of tigecycline (50 mg/kg) and a combination of tigecycline plus inosine (50 + 100 mg/kg) at left posterior gastropoda. Survival rates of larvae were recorded for 3 days.

### Statistical analyses

Statistical analysis was performed using GraphPad Prism 9.0 and SPSS software. All data were presented as means ± SD. Unless otherwise noted, unpaired *t* test between the two groups or one-way ANOVA among multiple groups were used to calculate *P* values (*P < 0.05, **P < 0.01, ***P < 0.001, ****P < 0.0001).

## Data Availability

Metabolomics data have been deposited in Mendeley Data (https://doi.org/10.17632/9xp4k5pb4v.1). RNA-sequencing data have been deposited in the National Center for Biotechnology Information (NCBI) Sequence Read Archive (SRA) database (PRJNA1137606).

## SUPPLEMENTAL MATERIAL

Supplemental material msystems.00797-24-s0001.pdf Supplemental figures and tables.

ASM does not own the copyrights to Supplemental Material that may be linked to, or accessed through, an article. The authors have granted ASM a non-exclusive, world-wide license to publish the Supplemental Material files. Please contact the corresponding author directly for reuse.
