# Supplementary material for: Inosine reverses multidrug resistance in Gram-negative bacteria carrying mobilized RND-type efflux pump gene cluster tmexCD-toprJ
Source: mSystems. 2024 Sep 10;9(10):e00797-24. doi: 10.1128/msystems.00797-24 (PMC11495011; doi:10.1128/msystems.00797-24)
Supplement: Supplemental material — Supplemental figures and tables. [file msystems.00797-24-s0001.pdf]

Supplementary Materials

Figures

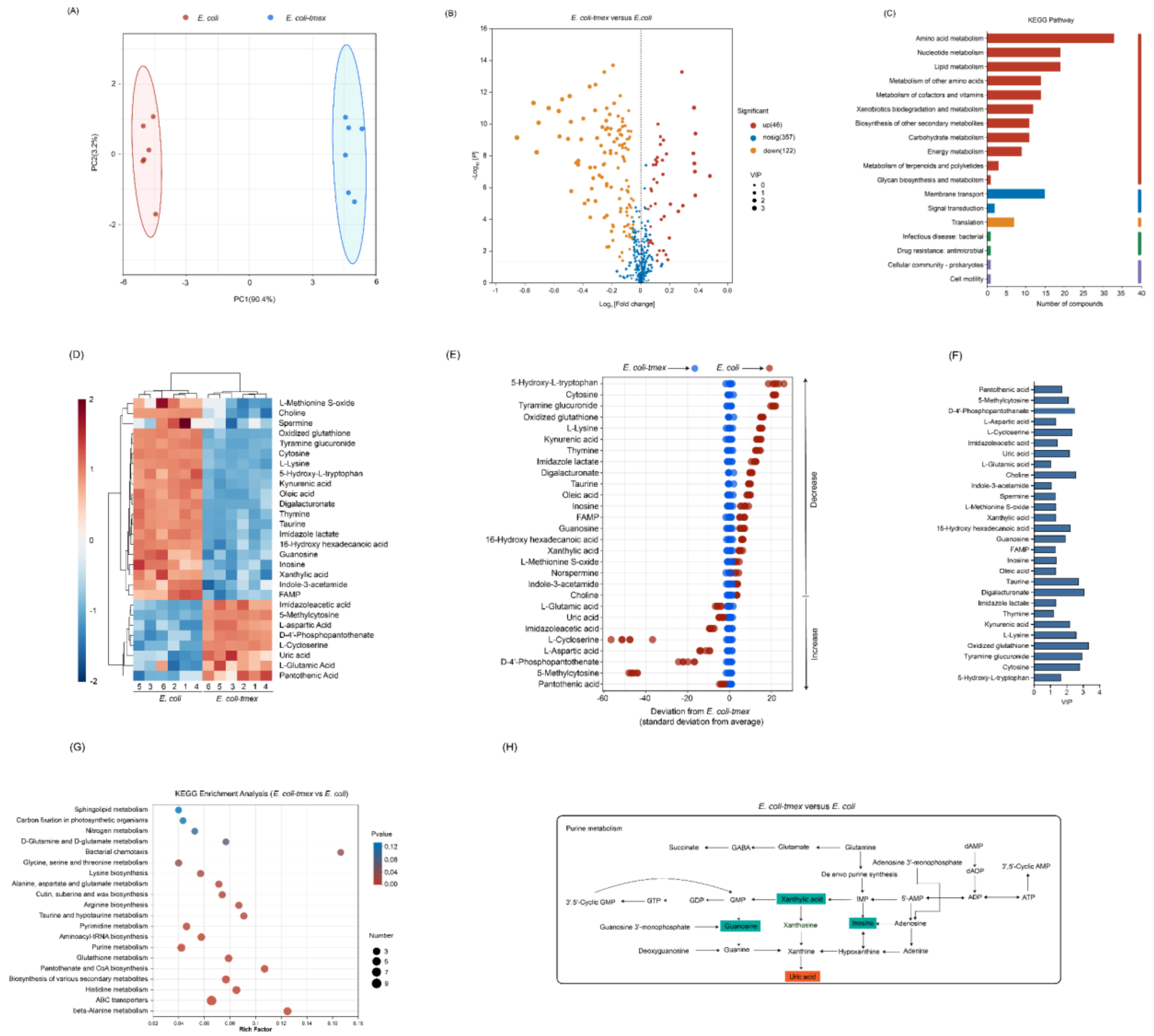

Figure S1. Metabolomic profiling of *tmexCD1-toprJ1*-negative and -positive *E. coli*.

(A) PCA of 168 metabolites identifies components 1 and 2 as determinants of variability in metabolite abundance in *E. coli-tmexCD1-toprJ1* and *E. coli*. **(B)** Volcano plot of metabolome analysis of *E. coli-tmexCD1-toprJ1* and *E. coli*. **(C)** Number of compounds enriched in KEGG pathways. **(D)** Heatmap showing relative abundance of differential metabolites in enriched in KEGG pathways. Blue to orange colors correspond to low to high abundance. FAMP, N-Formyl-4-amino-5-aminomethyl-2-methylpyrimidine. **(E)** Z scores (standard deviation from average) corresponding to data in (D). Each point represents one technical repeat in one metabolite. Green, *E. coli*; Orange, *E. coli-tmexCD1-toprJ1*. **(F)** The metabolite variable importance in the projection value (VIP), which indicates the contribution

of the metabolite to the difference between *E. coli-tmexCD1-toprJ1* and *E. coli*. **(G)** Enriched pathways in *E. coli-tmexCD1-toprJ1* ($p < 0.01$). **(H)** Scatter plots showing the abundance of downstream and upstream purine metabolites in *E. coli-tmexCD1-toprJ1*. **(I)** Metabolic regulation of *E. coli-tmexCD1-toprJ1*, superimposed on a metabolic network. Red, increased abundance of metabolites; Green, decreased abundance of metabolites; black, no difference of metabolites.

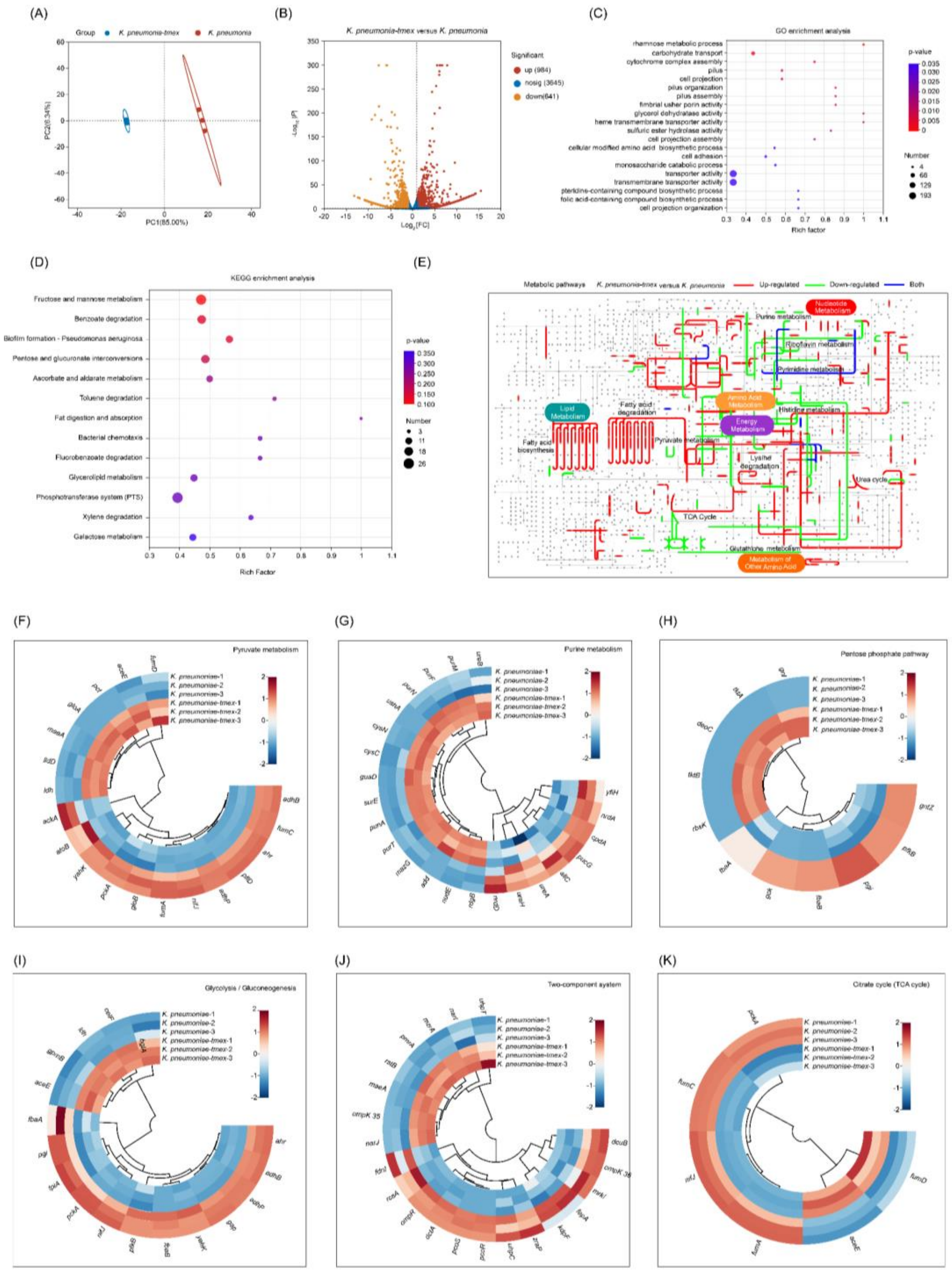

Figure S2. Transcriptomic analysis between *tmexCD1-toprJ1*-negative and -positive *K. pneumoniae.*

(A) Principal component analysis (PCA), **(B)** Volcano plot, **(C)** GO enrichment analysis, **(D)** KEGG enrichment analysis of the differential expression genes (DEGs) in *K. pneumoniae-tmexCD1-toprJ1*. **(E)** Integrated metabolic network in relation to differential genes by iPath. The red and blue are depicted the increased and decreased genes in *K. pneumoniae-tmexCD1-*

toprJ1. Selected DEGs involved in pyruvate metabolism **(F)**, purine metabolism **(G),** pentose phosphate pathway **(H),** glycolysis /gluconeogenesis **(I)**, Two-component system **(J),** and TCA cycle **(K)**.

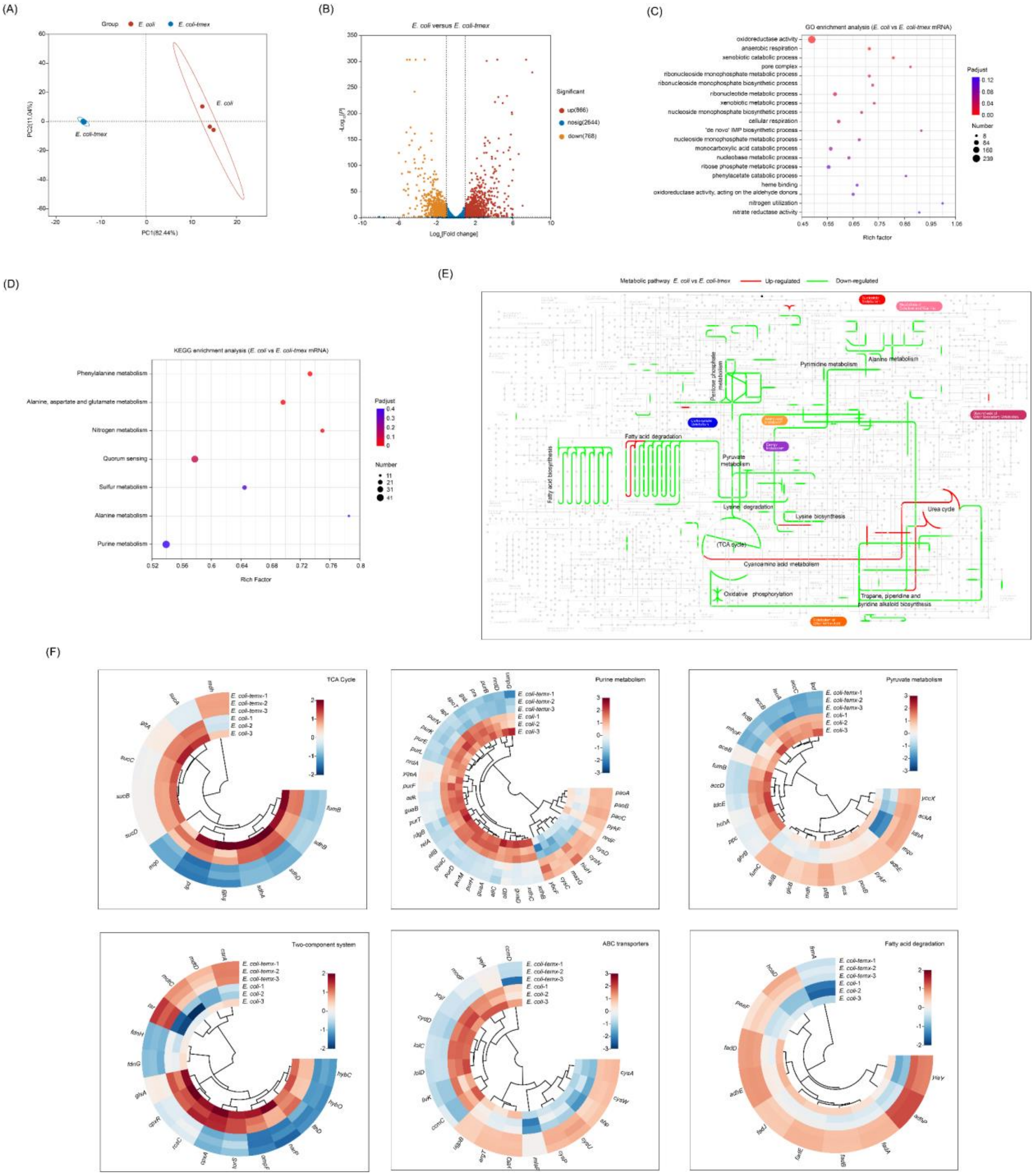

Figure S3. Transcriptomic analysis between *tmexCD1-toprJ1*-negative and -positive *E. coli.*

(A) Principal component analysis (PCA), **(B)** Volcano plot, **(C)** GO enrichment analysis, **(D)** KEGG enrichment analysis of the differential expression genes (DEGs) in *E. coli-tmexCD1-toprJ1*. **(E)** Integrated metabolic network in relation to differential genes by iPath. The red and blue are depicted the increased and decreased genes in *E. coli-tmexCD1-toprJ1*. Selected DEGs involved in TCA cycle **(F)**, purine metabolism **(G),** pyruvate metabolism **(H),** Two-component =system **(I)**, ABC transporters **(J),** and fatty acid degradation **(K)**.

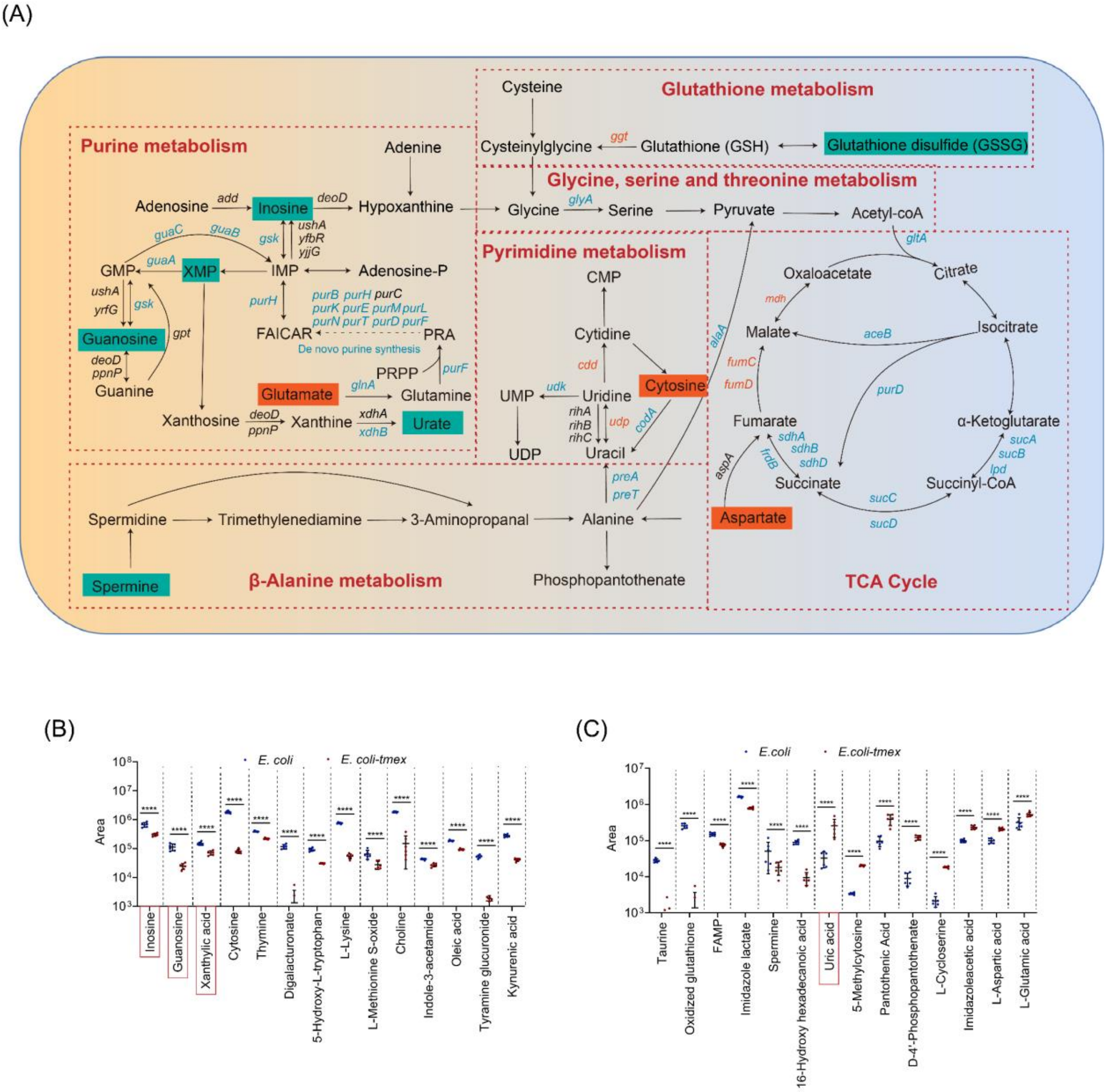

Figure S4. Map of differences metabolic in the purine metabolism between *E. coli-tmexCD1-toprJ1* and *E. coli*.

Upregulated and downregulated metabolites/genes are highlighted in red and green, respectively. **(B)** Scatter plots showing the abundance of downstream purine metabolites *in E. coli-tmexCD1-toprJ1*. **(C)** Scatter plots showing the abundance of upstream purine metabolites *in E. coli-tmexCD1-toprJ1*.

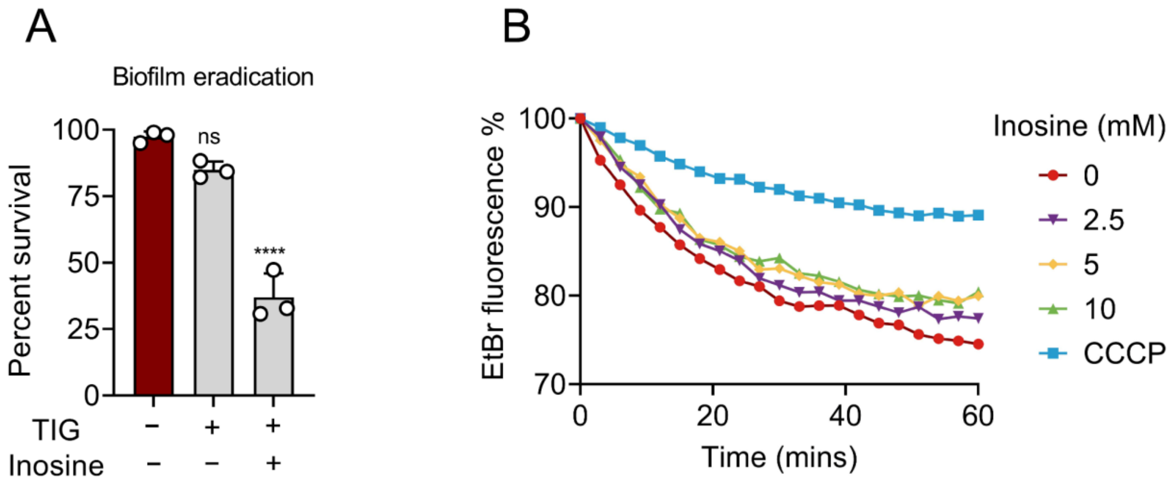

Figure S5. (A) Percent survival of *K. pneumoniae* RGF 140-1 biofilm under tigecycline treatment in the presence or absence of 20 mM inosine for 24 h. **(B)** The function of bacterial efflux pump in the presence of increasing concentrations of Inosine ranging from 0 to 10 mM.

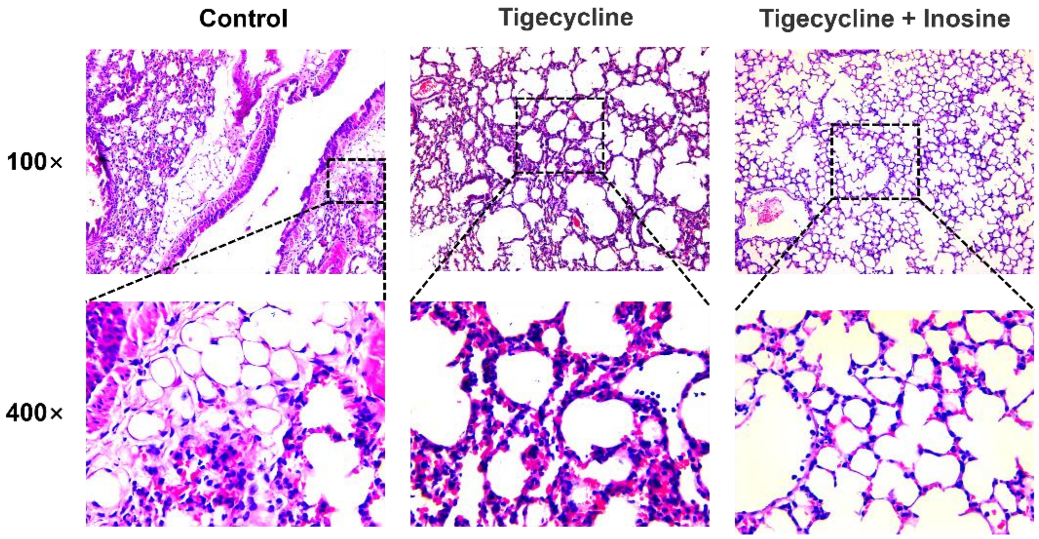

Figure S6. The lung histopathology of mice infected with *tmexCD1-toprJ1*-positive K. pneumoniae RGF 140-1 and treated with tigecycline alone or in combination with inosine.

Tables

Table S1. Minimum inhibitory concentration (MIC) analysis of tigecycline against different pathogens used in this study.

Bacteria and genotype	MIC (μg/mL)
E. coli DH5α	0.5
E. coli-pUC19-(*tmexCD1-toprJ1*)	8
K. pneumoniae K6	0.5
K. pneumoniae-pUC19-*(tmexCD1-toprJ1)*	16
K. pneumoniae 140-1-*(tmexCD1-toprJ1)*	32
K. pneumoniae- pUC19-*(tmexCD2-toprJ2)*	16
E. coli-pUC19-(*tmexCD3-toprJ3*)	8
P. mirabilis- pUC19-*(tmexCD3-toprJ3)*	16

Table S2. Antimicrobial susceptibility of *K. pneumoniae* RGF140-1 (*tmexCD1-toprJ1*).

Antibiotics	MIC (μg/mL)
Tigecycline	32
Tetracycline	>256
Oxytetracycline	>256
Gentamicin	128
Streptomycin	128
Tobramycin	8
Ceftiofur	16
Ceftazidime	8
Ciprofloxacin	16

Table S3. Primers for RT-qPCR analysis in this study.

Genes	Primers	Sequence (5'-3')
tmexC1	Forward	CCGTTACGAACCACTGGTGA
	Reverse	AGGATGGCGTTCTGGTTGAG
tmexD1	Forward	TACACCCTGATCCCGTCCAT
	Reverse	AGAGAAAGCCCATCAGCACC
toprJ1	Forward	TCTCACTTCATGCTCCGTCG
	Reverse	TCCAGGGTTTGTTCGACCAG
ompK 35	Forward	GGTTTACCATTGAGAGCAACGAC
	Reverse	AGCGGGTGTATTCATAGCGATAG
ompK 36	Forward	CCGTAGCTTACCTGCAGTCTAAA
	Reverse	TGAGACGATTTCGCCAGCAT
ompR	Forward	GGATGGTCTGCTGATTAAGTTCG
	Reverse	CTCCAGCAGGGCAATAATATAACC
EnvZ	Forward	CTGGGGATTTCGCTCTATTCCAA
	Reverse	CAGGGGAGCTTTTATTGACCTCT
csgD	Forward	GATGAATCGCGCTTAATTGAGGG
	Reverse	CGAGTTCTCTACTGGAGTCGATG
cpxA	Forward	GGAAGATCGCGAACAGATTTTCC
	Reverse	CTGAATAGCGGTTTCGACAATCG
cpxR	Forward	TTCACCCTGCTCTATCTGTTAGC
	Reverse	GGAAATATGCATATCGATGGCGC
	Reverse	AATACGTGCGACAACGACCT
